# Experimental Feeding of *Hydrilla verticillata* Colonized by Stigonematales Cyanobacteria Induces Vacuolar Myelinopathy in Painted Turtles (*Chrysemys picta*)

**DOI:** 10.1371/journal.pone.0093295

**Published:** 2014-04-02

**Authors:** Albert D. Mercurio, Sonia M. Hernandez, John C. Maerz, Michael J. Yabsley, Angela E. Ellis, Amanda L. Coleman, Leslie M. Shelnutt, John R. Fischer, Susan B. Wilde

**Affiliations:** 1 D. B. Warnell School of Forestry and Natural Resources, University of Georgia, Athens, Georgia, United States of America; 2 Southeastern Cooperative Wildlife Disease Study (SCWDS), Department of Population Health, Wildlife Health Building, College of Veterinary Medicine, University of Georgia, Athens, Georgia, United States of America; 3 The Athens Veterinary Diagnostic Laboratory, College of Veterinary Medicine, University of Georgia, Athens, Georgia, United States of America; 4 The University of Georgia College of Veterinary Medicine, University of Georgia, Athens, Georgia, United States of America; INIAV, I.P.- National Institute of Agriculture and Veterinary Research, Portugal

## Abstract

Vacuolar myelinopathy (VM) is a neurologic disease primarily found in birds that occurs when wildlife ingest submerged aquatic vegetation colonized by an uncharacterized toxin-producing cyanobacterium (hereafter “UCB” for “uncharacterized cyanobacterium”). Turtles are among the closest extant relatives of birds and many species directly and/or indirectly consume aquatic vegetation. However, it is unknown whether turtles can develop VM. We conducted a feeding trial to determine whether painted turtles (*Chrysemys picta*) would develop VM after feeding on *Hydrilla* (*Hydrilla verticillata*), colonized by the UCB (*Hydrilla* is the most common “host” of UCB). We hypothesized turtles fed *Hydrilla* colonized by the UCB would exhibit neurologic impairment and vacuolation of nervous tissues, whereas turtles fed *Hydrilla* free of the UCB would not. The ability of *Hydrilla* colonized by the UCB to cause VM (hereafter, “toxicity”) was verified by feeding it to domestic chickens (*Gallus gallus domesticus*) or necropsy of field collected American coots (*Fulica americana*) captured at the site of *Hydrilla* collections. We randomly assigned ten wild-caught turtles into toxic or non-toxic *Hydrilla* feeding groups and delivered the diets for up to 97 days. Between days 82 and 89, all turtles fed toxic *Hydrilla* displayed physical and/or neurologic impairment. Histologic examination of the brain and spinal cord revealed vacuolations in all treatment turtles. None of the control turtles exhibited neurologic impairment or had detectable brain or spinal cord vacuolations. This is the first evidence that freshwater turtles can become neurologically impaired and develop vacuolations after consuming toxic *Hydrilla* colonized with the UCB. The southeastern United States, where outbreaks of VM occur regularly and where vegetation colonized by the UCB is common, is also a global hotspot of freshwater turtle diversity. Our results suggest that further investigations into the effect of the putative UCB toxin on wild turtles *in situ* are warranted.

## Introduction

Vacuolar myelinopathy (VM) is a neurologic syndrome that primarily affects birds associated with freshwater habitats. The effects of VM on wild birds are documented for American coots (*Fulica americana*), bald eagles (*Haliaeetus leucocephalu*s), mallards (*Anas platyrhynchos*), ring-necked ducks (*Aythya collaris*), buffleheads (*Bucephala albeola*), Canada geese (*Branta canadensis*), great horned owls (*Bubo virginianus*), and killdeer (*Charadrius vociferus*) in approximately 20 southeastern U.S. reservoirs ranging from Texas to North Carolina [1], [3]–[5]. It is thought that birds develop VM by directly or indirectly consuming aquatic vegetation colonized by a novel species of epiphytic cyanobacteria in the order Stigonematales (hereafter “UCB” for “uncharacterized cyanobacterium”) that produces a yet to be described toxin(s) [6], [7]. The UCB grows in high abundance on *Hydrilla* (*Hydrilla verticillata*), a widespread invasive exotic plant, although it can also grow on several native aquatic plant species [6]. Birds may acquire the toxin(s) directly by ingesting plants that are colonized with the UCB or indirectly by feeding on herbivorous prey such as invertebrates [8] or other bird species that have fed on plants that are colonized with the UCB [9]. Affected birds develop microscopic vacuoles in the white matter of the central nervous system. Lesions tend to be most prominent in the optic tectum but can occur in the cerebrum, cerebellum, brain stem, or spinal cord. Degenerative lesions in peripheral nerves have rarely been reported. Ultrastructurally, vacuolation is due to splitting of myelin lamellae at the intraperiod line, consistent with intramyelinic edema. These lesions result in variable neurologic dysfunction that in severe cases can result in death within a few days [2]–[4], [10].

A number of studies stress the need to evaluate the risk that consumption of vegetation colonized by the UCB poses to other taxa [11], [12]. Previous work showed that grass carp (*Ctenopharyngodon idella*) experimentally fed *Hydrilla* colonized with the UCB developed vacuolations consistent with avian models, yet domestic pigs (*Sus domesticus*) and laboratory mice did not [5], [13], [14]. To date, there are no reports for any species representing the remaining major vertebrate lineages (amphibians or reptiles).

Freshwater turtles have a number of characteristics that, if susceptible to the putative UCB toxin(s), make them likely candidates to develop vacuolar myelinopathy. Turtles and crocodilians are members of the Archosauria and therefore are the closest extant relatives to birds [15]. The southeastern United States, the current location of VM outbreaks, is a global hotspot of freshwater turtle diversity with ∼10% of the world's turtle species occurring in the region [16]. The vast majority of these turtles occur in freshwater, many species are omnivorous or herbivorous, and several feed extensively on submerged aquatic vegetation including *Hydrilla*, or on invertebrates that graze on epiphytic algae [17]; and turtles are known to be susceptible to other food chain-linked cyanotoxins [18]. Therefore, the objective of this study was to test the hypothesis that turtles fed *Hydrilla* colonized by the UCB and verified to be neurotoxic to birds would develop clinical signs of neurologic disease and histologic lesions similar to those of described in birds with vacuolar myelinopathy.

### Focal turtle species

We selected painted turtles (*Chrysemys picta*) as our focal species for this study. Painted turtles are one of the most thoroughly studied turtle species in the world, and their husbandry protocols are well established [17], [19]. They are abundant and readily available in regions where they occur and adapt well to captivity [20]. As a member of Emydidae, they are related to most of the turtle species in the southeastern U.S., and they are omnivorous but highly herbivorous as adults. We have documented *Hydrilla* in the gut contents of painted turtles in reservoirs experiencing VM epornitics (Mercurio et al. unpublished data) and they are known to feed on invertebrates that graze on epiphytic algae [17].

## Materials and Methods

### Ethics statement

All procedures were approved by the University of Georgia's Institutional Animal Care and Use Committee (A2012 02-001-Y2-A4). Field studies did not involve endangered or protected species and wildlife collections were permitted by the U.S. Fish and Wildlife Service (MB779238-0) and the Georgia Department of Natural Resources (29-WBH-12-95), which allow for the collection of wildlife from Georgia public state lands. In addition, the Henry County Water and Sewerage Authority provided permission to access Upper Towaliga Reservoir (33.3472°,−84.2145°). The University of Georgia Golf Course permitted access to a pond in Athens, GA (33.9041°, −83.3674°).

### First *Hydrilla* collection

Approximately enough *Hydrilla* to fill three, 50 gallon coolers was collected from J. Strom Thurmond Reservoir (33.6972°,−82.2540°) on the border of Georgia and South Carolina during a VM epornitic and Lake Seminole on the Georgia-Florida border in December 2011. *Hydrilla* was transported within clean zip top plastic bags (3.79 l) on ice to the University of Georgia [12]. VM positive birds have been recovered in late fall from J. Strom Thurmond Reservoir annually since 1998 and *Hydrilla* in this reservoir is consistently colonized by the UCB [1], [6]. Lake Seminole has never experienced a VM case, and the UCB has never been detected at this site [6]. Light and epifluorescent microscopy were used to confirm the presence/absence of the UCB following established methods [6], [7]. Briefly, 5 representative leaves were wet mounted on a glass slide. Light microscopy and a rhodamine filter set were used to visualize cyanopigments on *Hydrilla* leaves and the presence/absence of the UCB colonies were documented via visual assessment. The rest of the *Hydrilla* was frozen at −20°C for 48 hours. To lyophilize the material, one gallon sized paper bags of frozen *Hydrilla* were then placed in the lyophilizing chamber of a Labconco Freeze Dryer 5 (Labconco, Kansas City, MO) at ∼5 mm Hg for 48 hours or until completely dry. Once dry, *Hydrilla* was stored in sealed plastic bags in a temperature controlled facility at 26.6°C.

### Validation of the toxicity of the first *Hydrilla* collection

Because some plant samples with the UCB do not induce VM when consumed, a feeding trial was conducted to determine the toxicity of the *Hydrilla* [21]. Domestic chickens (*Gallus gallus domesticus*) are susceptible to VM through dietary exposure of aquatic vegetation collected from sites where VM has been documented in wild birds [5]. A chicken feeding trial was conducted as previously described with the first collection of *Hydrilla* to assess its potential to induce VM [5]. Briefly, 4-week-old specific pathogen free leghorn chickens (0.8–1.5 kg, n = 10) were housed at the University of Georgia Poultry Diagnostic and Research Center in Horsfal (isolation) units. Once a week, chickens were weighed and received a full physical and neurologic exam consistent with previous trials [5], [22], [23]. Mentation, posture, attitude, movement, gait, postural reactions, spinal reflexes, and cranial nerve function were assessed. Limbs were palpated to evaluate asymmetry, masses, tenderness, contour, and tone. Birds were also weighed and observed for any superficial injuries. Chickens were allowed to acclimate to laboratory conditions for four days and were fed a non-medicated starter feed produced by the University of Georgia feed mill *ad libitum* (∼30 g/kg bw/day) out of ceramic bowls. All chickens were in good body condition and no physical or neurologic abnormalities were noted at the beginning of the trial. Chickens were then randomly assigned to two treatment groups. Five treatment group birds were fed 30 g/kg bw/day of poultry starter feed and 2 g/kg bw/day of lyophilized *Hydrilla* colonized with the UCB from J. Strom Thurmond Reservoir for 28 days. The other five control group birds were fed the same volume of poultry starter feed and lyophilized *Hydrilla* free of the UCB from Lake Seminole. Each bird was monitored twice daily for clinical signs of VM (difficulty standing or ambulating, ataxia, loss of balance, limb paresis and/or head droop) and were weighed twice a week [10].

All chickens were humanely euthanized on day 28 with CO_2_ followed by cervical dislocation. Calvaria were opened and partially removed with rongeurs to expose the dorsal surface of the brain. Brains were removed intact from the calvaria using a scalpel and/or scissors and immediately placed into 10% neutral buffered formaldehyde. Following ten days of fixation, brains were halved longitudinally. A single longitudinal section was then made 1–2 mm lateral to midline and an additional 1–2 transverse sections were made through the optic lobe. Resulting sections were placed whole into a cassette. These sections were routinely processed, embedded in paraffin, sectioned at 5 μm, and stained with hematoxylin and eosin prior to light microscopic examination by a veterinary pathologist [3]. All treatment chickens fed *Hydrilla* from J. Strom Thurmond Reservoir were bright, alert, responsive and eating well throughout the entire trial but developed very mild neurologic clinical signs (mild ataxia beginning on day seven until the end of the trial) and developed vacuolations consistent with VM, whereas none of the control birds fed *Hydrilla* from Lake Seminole developed clinical signs or vacuolations.

In December 2012, we collected more fresh *Hydrilla* (same volume) from Upper Towaliga Reservoir in Henry County, GA and from Lake Seminole (30.7428°,−84.8776°). Like J. Strom Thurmond Reservoir, Upper Towaliga Reservoir undergoes annual VM outbreaks and *Hydrilla* in this reservoir is routinely colonized by the UCB. This *Hydrilla* was collected during a VM epornitic at Upper Towaliga Reservoir and was transported on ice to the University of Georgia as previously described. The presence/absence of the UCB was verified via light and epifluorescent microscopy as previously described. VM lesions were verified in coots recovered from Upper Towaliga Reservoir during the fall of 2012 (Fischer et al. unpublished data). *Hydrilla* was frozen at−20°C in zip top plastic bags (3.79 l) and was thawed as needed.

### Turtle feeding trial

Adult painted turtles (*Chrysemys picta*, straight carapace length >7 cm: 7 females, 3 males) were collected using canned sardine (Crown Prince, City of Industry, CA) baited hoop traps (model TN210; Memphis Net and Twine Co, Inc., Memphis, TN) from a pond in Athens, GA where an VM outbreak has never been documented and the UCB has never been documented. Turtles were individually transported in clean plastic bins to the Whitehall Herpetology Laboratory, a climate controlled facility, where we completed physical and neurologic exams as described below. The ten turtles were selected from a larger sample and were determined to be neurologically and physically normal. The turtles were housed individually in 37.8 l (50.8 cm×25.4 cm×30.48 cm) glass tanks following standard husbandry protocols [19]. Briefly, incandescent lights provided a 12 hour light cycle, the ambient temperature was maintained in the room at 26.6°C, water temperature at 24.4°C, and basking surfaces (clay bricks) at ∼32.2°C. Fresh city water was supplied as needed to maintain a depth of 20 cm. Water quality was maintained using aquarium filters (Fluval Nano; Rolf C. Hagen Corp, Mansfield, MA). Each week the water was removed, the gravel was rinsed, the tank was scrubbed with a mild dish detergent and rinsed thoroughly, one half of the old water was replaced, and the tank was filled up to 20 cm with fresh water. Turtles were monitored daily for gross appearance, behavior, food consumption, mentation, and were allowed to acclimate to laboratory conditions and to our feeding delivery method for a minimum of 15 days. During this time they were fed ReptoMin floating turtle sticks (Spectrum Brands Inc., Melle, Germany) homogenized into a uniform powder and packed into transparent gelatin capsules (Capsuline Corporation, Pompano Beach, FL and Torpac Inc., Fairfield, NJ) at 0.02 kcal/g body weight/day, calculated using an allometric food calculator developed by the University of Georgia College of Veterinary Medicine Teaching Hospital [24].

Five turtles were randomly assigned into either a treatment *Hydrilla* or a control *Hydrilla* group. *Hydrilla* was fed to turtles in two ways to maximize consumption. The *Hydrilla* from the first collection with confirmed UCB toxicity status was packed into transparent gelatin capsules. To increase palatability and provide additional nutrition, capsules were coated in a mixture of sardine oil and ReptoMin prior to feeding. Each turtle was offered ∼6 g/kg bw/day of their assigned *Hydrilla* diet in floating gelatin capsules. ReptoMin was also provided as needed to maintain each turtle's body weight relative to the start of the study. Starting on day 30 of the feeding trial, 50 g of intact floating *Hydrilla* from the second collection with confirmed UCB toxicity status was added to each tank each day to maximize *Hydrilla* consumption by turtles. The amount of the floating *Hydrilla* consumed each day was measured to the nearest 10 g. More accurate monitoring of material consumed was not possible because the turtles shredded *Hydrilla* during normal feeding activities.

### Physical and neurologic exams

Turtles underwent a complete physical and neurologic exam once per week. Turtles were weighed and were observed for any obvious injuries, lesions, dysecdysis, or abrasions. A neurologic exam as described for reptiles in [25] and [24] was performed to assess the mental status, attitude, general activity, head and body posture, limb movement and coordination, gait, position in the water while swimming, and sensory and motor responses. Briefly, the turtles were first observed from a distance within their tanks for coordination while swimming, posture, and mentation prior to handling. The turtles were then removed from their tanks and held by the observer. The limbs were palpated to determine musculoskeletal symmetry, tone, strength, and tenderness. Reflexes were described as absent, reduced, normal, or clonus (where applicable) unless otherwise stated. Leg and head withdrawal reflexes and the ability to maintain their head position in a horizontal plane while rotated and listed in midair were assessed. The function of cranial nerves II, IV, and VII was assessed by inciting a menace response in a standard manner by obscuring the vision in one eye and making a slow threatening hand gesture to the other eye [24]. Cranial nerves III, IV, and VI functions were assessed by observing for strabismus (present/absent). Cranial nerve V function was evaluated by assessing mandibular movement during feeding (normal/abnormal). Cranial nerve VIII function was assessed by observing for nystagmus by moving the turtle's head side to side in a horizontal plane and observing the resulting movement of eyes. The presence/absence of nystagmus when the turtle was held stationary was also assessed. Cranial nerve VIII function was also assessed by observing for head tilting, rolling, and the righting reflex. The function of cranial nerves IX, XI, and XII was assessed by looking for signs of dysphagia. The turtles were then allowed to ambulate to evaluate symmetry of movement, gait, and posture.

### Detection of vacuolations

At the conclusion of the trial (97 days) or if an individual developed neurologic signs, humane euthanasia was performed using an injection of sodium pentobarbital (100 mg/kg) [24] with a 22 gauge needle into the subcarapacial vein followed immediately by complete necropsy. Briefly, the plastron was removed using a striker saw to expose the coelomic cavity. Representative samples of liver, lungs, kidney, heart, spleen, gonads, stomach, and intestine were excised and placed into 10% neutral buffered formalin. An approximately 5 mm segment of skeletal muscle and peripheral nerve was excised from a rear leg. Brains were removed in a fashion similar to that previously described for the chickens with the exception that the proximal 2–5 mm of the spinal cord was also removed with the brain. The brain was halved longitudinally and halves were immediately placed into fixative (either 10% buffered formaldehyde for histopathology or chilled 2% glutaraldehyde, 2% paraformaldehyde, and 0.2% picric acid in a 0.1 M cacodylate buffer (pH 7.2) for transmission electron microscopy (EM). Following a fixation time of approximately 30 days, the formalin fixed half of the brain was sectioned transversely at approximately 2 mm intervals, resulting in 5 total sections that were placed into a divided cassette. Spinal cord was also sectioned transversely, resulting in 3–5 sections that were placed into a second cassette. Formalin fixed tissues were routinely processed, embedded, and stained as previously described for the chickens and were subsequently examined by a pathologist with experience in chelonian histopathology following previously described methods [9]. Subsequently one treatment and one control turtle were randomly selected for EM examination at the Electron Microscopy Laboratory at the University of Georgia [5]. Transmission electron microscopy specimens were post-fixed in 1% osmium tetroxide, serially dehydrated, infiltrated in an acetone/epoxy plastic, and then embedded in a plastic mold. Plastic blocks were cut with an ultramicrotome, and thick sections were stained with toluidine blue to identify optimal areas for thin sectioning. Thin sections were cut at 55–60 nm, placed on copper grids, and stained with uranyl acetate and lead citrate.

### Statistical analysis

A Student's t-test for paired samples was used to determine if all turtles increased in weight from the beginning of the trial to the end. A Student's t-test was then used to determine if the change in weight over time significantly varied between treatment groups. An Analysis of Covariance was also used to determine if the average amount of *Hydrilla* consumed per day varied between treatment groups while accounting for body mass [26]. Statistical analyses were completed in IBM SPSS Version 21.

## Results

### Turtle feeding trial

The weight of the turtles increased significantly throughout the trial (t _α 0.05, 9_ = −2.788, p = 0.021) with no difference between the treatment and control groups (t _α 0.05, 7_ = −0.030, p = 0.977). Control turtles consumed an average of 2.76 g of lyophilized *Hydrilla*/kg bw/day (SE±0.73 g) and turtles fed *Hydrilla* with UCB consumed an average of 1.58 g of lyophilized *Hydrilla*/kg bw/day (SE±0.13 g). The main effect of treatment group was not significant, F(1,6) = 0.65, p = 0.45, ηp^2^ = 0.10, nor was body mass, F(1,6) = 0.52, p = 0.49, ηp^2^ = 0.08, nor was the interaction between mass and treatment group, F(1,6) = 0.39, p = 0.55, ηp^2^ = 0.06. For floating *Hydrilla*, control turtles consumed an average of 14.5 g/kg bw/day (SE±3.22 g) whereas turtles fed *Hydrilla* with UCB consumed an average of 8.55 g/kg bw/day (SE±1.03 g). The main effect of treatment group was not significant, F(1,6) = 1.11, p = 0.33, ηp^2^ = 0.156, nor was body mass, F(1,6) = 0.27, p = 0.63, ηp^2^ = 0.04, nor was the interaction between mass and treatment group, F(1,6) = 0.71, p = 0.43, ηp^2^ = 0.11.

All turtles appeared healthy until day 82 of the trial. Between days 82–89, the five treatment turtles began displaying neurologic dysfunction, including, but not limited to, various degrees of ataxia (mild gait asymmetry to severe limb dragging- Video S1) and inability to right themselves, in addition to performing poorly on one or more aspects of the neurologic exam (Table 1). Three of the turtles were euthanized on day 82. Two turtles (#85 and 119) only displayed mild neurologic deficits on day 89, the first observation of deficits. We maintained these turtles, which were still alert and eating well, until day 97 to observe the progression of clinical signs. During this time, the feeding volume decreased to anorexia in #85, however, its gait improved to normal by day 94. Turtle 119 continued to eat and intermittently displayed mild neurologic deficits. Both turtles were subsequently euthanized on day 97. Control turtles appeared healthy throughout the entire trial.

**Table 1:** Clinical signs observed in the treatment group turtles after the first observed deficits on day 82 of the experiment.

ID #	Anorexic?	Gait and movement Normal?	Able to Swim?	Mentation	Spinal and other Reflexes Normal?	Could keep head in horizontal plane when rotated and listed?
107	Yes	Would not ambulate	Floating upside down	Stupor	No attempt to right itself	Yes
104	Yes	Ataxia	No	Stupor	Unable to right itself	Reduced ability
118	No	Ataxia	Yes	Depressed	No head withdrawal, no attempt to right itself	Reduced ability
85	Yes	Ataxia	Yes	Alert	Unable to right itself and head withdrawal reflex was reduced	Yes
119	No	Ataxia	Yes	Alert	Unable to right itself	Yes

### Diagnostic findings

**Figure 1:**
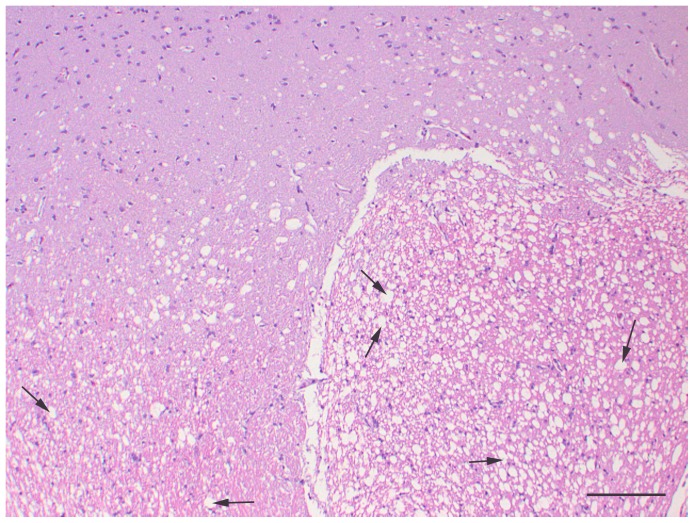
Histopathological slide of the optic tectum of a painted turtle fed toxic *Hydrilla* material. Painted turtle (*Chrysemys picta*), brain: Numerous clear vacuoles (black arrows) representing myelin degeneration and dilation of axonal sheaths are present in the white matter of a turtle treated with toxic hydrilla. H&E, 100X. Scale bar is 100 μm.

**Figure 2:**
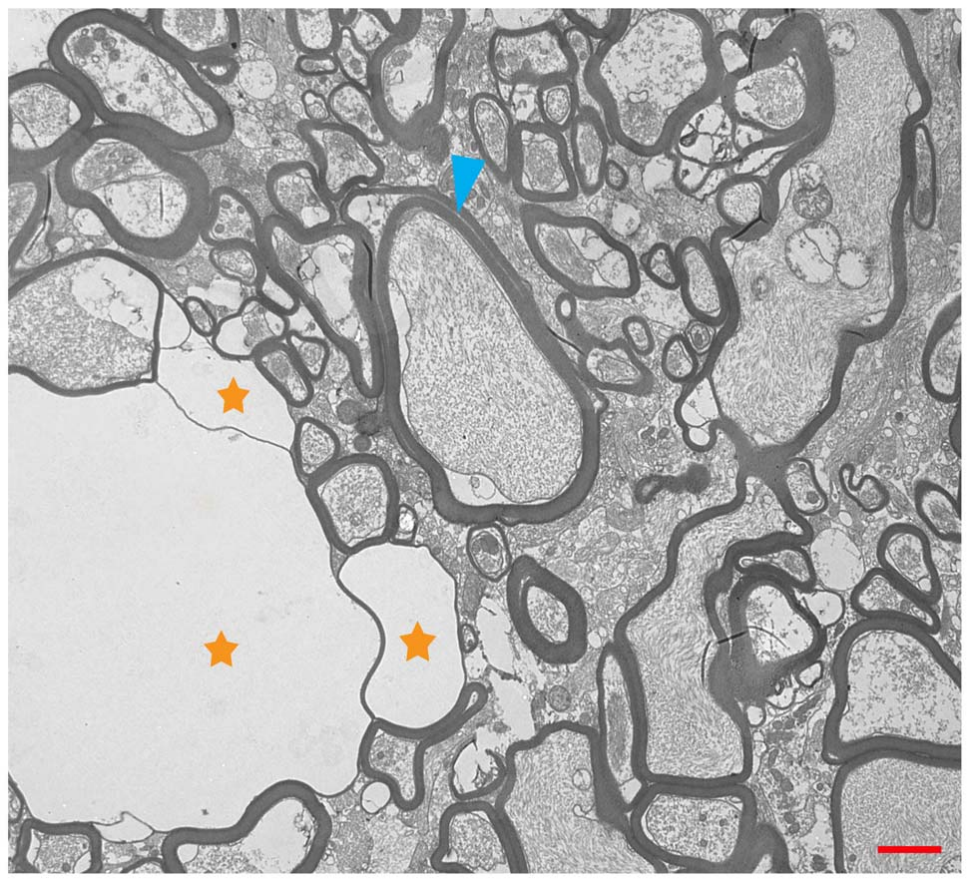
Electron Micrograph of central nervous tissue of a painted turtle fed toxic *Hydrilla* material. Electron Microscopy, painted turtle (*Chrysemys picta*), brain: Axons are swollen and degenerate and myelin sheaths are frequently disrupted by large, clear, intramyelinic vacuoles (orange stars). In less severely affected axons, splitting can be seen to occur at the intraperiod lines (blue arrow). Scale bar is 2 μm.

**Figure 3:**
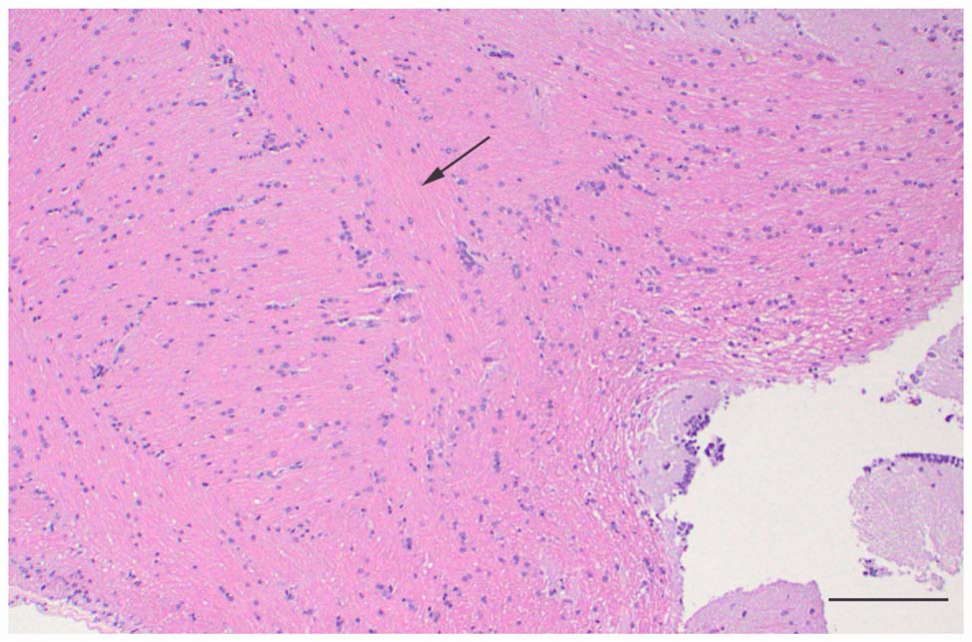
Histopathological slide of the optic tectum of a normal turtle. Painted turtle (*Chrysemys picta*), brain: white matter, indicated by black arrows, appears normal with no evidence of vacuolation or myelin degeneration. H&E, 100X. Scale bar is 100 μm.

No gross abnormalities were observed for any turtles at necropsy. All turtles were in good body condition and contained food in the gastrointestinal tract, with the exception of the three turtles in the treatment group that displayed anorexia or reduced feed intake towards the end of the trial. Significant histologic abnormalities were not observed in any of the controls. However, all turtles in the treated group had severe, diffuse vacuolation of white matter throughout the entire brain, including cerebrum, cerebellum, and brain stem, and spinal cord with no single area appearing to be consistently more or less affected than other areas. Mild multifocal inflammatory lesions consisting of lymphocytic perivascular cuffing were noted in peripheral nerves. However, these were present and similar in both the control and treatment groups and may have been related to schistosomes which were an incidental finding in several turtles. Significant lesions were not present in any of the other examined organs (kidney, liver, heart, lung, spleen, gonad, and gastrointestinal tract) in both treatment groups. Light microscopic changes were present throughout the white matter of the brain and spinal cord of treatment group turtles and consisted of coalescing, round to ovoid, clear vacuoles that were approximately 5–40 μm in diameter (Figure 1). Similar but less widespread vacuolation was also noted in the Purkinje and inner granular cell layers of the cerebellum. However, perikarya were unaffected. In the cerebral gray matter, scattered vacuoles, either individually or in small clusters, were also observed but this tended to occur at white matter interfaces. Electron microscopic findings in the brain of the treatment group turtle consisted of axonal swelling and degeneration with splitting of myelin at the intraperiod line (Figure 2). No significant histologic (Figure 3) or electron microscopic abnormalities were noted in the brain of the control turtles.

## Discussion

Our study demonstrates that a common freshwater turtle species, the painted turtle, can develop neurologic signs and vacuolations consistent with VM from consuming *Hydrilla* with UCB. Clinical signs in turtles were consistent with avian models, presenting as varying degrees of neurologic and physical impairment [5], [10]. A subjective attempt was made to correlate lesions with neurologic severity and/or type of neurologic signs. However, with the exception of the cerebellar lesions, all affected turtles appeared to have similar, severe, widespread lesions and no such correlations could be identified. While some variation in distribution and severity was present among the cerebellar lesions, this did not appear to correlate with any differences in the clinical signs. These findings are similar to those described in birds with VM [3], [10].

Though the specific agent or agents that cause VM have not been identified, we believe that our results provide strong evidence that the same active agent(s) that induce VM in birds and are associated with ingestion of the UCB induce the lesions and associated neurologic disease in painted turtles. There might be a generalized effect among these two closely related taxa. A previous study demonstrated that grass carp fed toxic *Hydrilla* also developed vacuolations, suggesting the toxin(s) produced by the UCB may have broad neurologic effects among vertebrates [10]. Although two studies of domestic pigs and one study of laboratory mice fed toxic *Hydrilla* failed to find evidence of neurologic signs or detectable vacuolations, the authors of those studies emphasize the dose and/or duration of toxin(s) exposure may vary among taxa and experimental design, and may not have been sufficient to induce disease [5], [13], [14]. Our results support the hypothesis that taxa may vary in the required dosage or exposure duration to induce neurologic lesions. Standard avian trials are less than 30 days in length, and birds are often symptomatic within a few days. Grass carp euthanized 37 days post exposure to colonized *Hydrilla* had vacuolations, although no clinical signs were noted [10]. In our study, chickens fed *Hydrilla* colonized by the UCB exhibited mild neurologic signs within 7 days; however, turtles fed the same *Hydrilla* did not exhibit detectable clinical signs until 82 days. Possible explanations for this difference are the slower metabolism of ectotherms when compared to endotherms, differences in digestive efficiency, different metabolic pathways, an innate resistance to the toxin, or some other unknown factor.

We caution that while turtles may be sensitive to the UCB toxin, it remains to be determined whether turtle populations are vulnerable to the UCB's spread and invasions of freshwaters. Vulnerability incorporates both sensitivity and exposure. Many ponds and reservoirs in the southeastern U.S. have dense *Hydrilla* or native submerged aquatic vegetation that supports abundant concentrations of the UCB [1], [6]. In those systems, a diet consisting of large amounts of *Hydrilla* may be biologically realistic, particularly for highly herbivorous turtles (e.g., *Trachemys* and *Pseudemys* spp.) [27], [28]. However, VM epornitics occur during late fall-winter, leading some to suggest that toxin production is related to season [2]. Most turtle species in the southeastern U.S. exhibit limited activity in the late fall to winter and may limit feeding during the cooler months of peak VM epornitics. To date, no large-scale die offs of aquatic turtles have been reported in reservoirs where VM die offs were reported for birds. Dead turtles may sink, decompose, or become scavenged in the water, which may contribute to low detection rates of impaired turtles. Moreover, our observations were that impaired turtles could show some motor recovery despite significant lesions in the brain. Turtles that have lesions but are not clearly distressed may not be reported [9], and the dominant effects of ingesting the UCB may be subacute and not associated with high mortality. It is also not known whether turtles can recover longer term from the neurologic damage associated with ingesting the UCB. Clearly, more studies will be needed to elucidate important details on the epidemiology and vulnerability of the UCB to turtles and other wildlife. We propose a near term need for sensitivity studies of wider suites of taxa including those feeding directly or indirectly on UCB host plants, and studies of the seasonality of toxin production relative to seasonal variation in foraging rates of exposed taxa to determine potential population level vulnerabilities.

## Supporting Information

Video S1 **Ataxia (gait asymmetry and limb dragging) displayed by a painted turtle (*Chrysemys picta*) fed toxic *Hydrilla* material.**
(MOV)
